# Ego Depletion Impairs Implicit Learning

**DOI:** 10.1371/journal.pone.0109370

**Published:** 2014-10-02

**Authors:** Kelsey R. Thompson, Daniel J. Sanchez, Abigail H. Wesley, Paul J. Reber

**Affiliations:** 1 Department of Psychology, Northwestern University, Evanston, Illinois, United States of America; 2 Computer Science Laboratory, SRI International, Menlo Park, California, United States of America; Utrecht University, Netherlands

## Abstract

Implicit skill learning occurs incidentally and without conscious awareness of what is learned. However, the rate and effectiveness of learning may still be affected by decreased availability of central processing resources. Dual-task experiments have generally found impairments in implicit learning, however, these studies have also shown that certain characteristics of the secondary task (e.g., timing) can complicate the interpretation of these results. To avoid this problem, the current experiments used a novel method to impose resource constraints prior to engaging in skill learning. *Ego depletion* theory states that humans possess a limited store of cognitive resources that, when depleted, results in deficits in self-regulation and cognitive control. In a first experiment, we used a standard ego depletion manipulation prior to performance of the Serial Interception Sequence Learning (SISL) task. Depleted participants exhibited poorer test performance than did non-depleted controls, indicating that reducing available executive resources may adversely affect implicit sequence learning, expression of sequence knowledge, or both. In a second experiment, depletion was administered either prior to or after training. Participants who reported higher levels of depletion before or after training again showed less sequence-specific knowledge on the post-training assessment. However, the results did not allow for clear separation of ego depletion effects on learning versus subsequent sequence-specific performance. These results indicate that performance on an implicitly learned sequence can be impaired by a reduction in executive resources, in spite of learning taking place outside of awareness and without conscious intent.

## Introduction

Implicit learning is defined as the ability to extract statistical covariation from the environment over experience and use this to guide and improve behavior [1], [2]. The implicit learning process occurs outside of awareness or intention to learn, leading to improved performance without conscious knowledge of the information used or the prior process of acquisition. Implicit learning and the performance of an implicitly acquired skill are often described as “automatic” and unaffected by constraints to central processing resources [3], though there has been much debate about whether this is indeed the case.

A wide range of studies have examined the effects of imposing resource constraints during implicit learning using dual task methodology (e.g., [4], [5], [6], [7]) and the classic SRT (Serial Reaction Time) implicit learning task. Many of these studies observed apparent reductions in the rate of implicit learning under dual task conditions, but it has also been frequently observed that the specific timing of the secondary task has a major impact on the measurement of learning. Frensch, Lin, and Buchner [8] and Hsiao and A.S. Reber [9] reported that if the processing demands of the secondary task occur simultaneously with a serial choice reaction time response, it is possible to disrupt the expression of knowledge even though learning is continuing. However, Schumacher and Schwarb [6] found that simultaneous task demands reduced learning only when these constrained central processing resources. Jiménez and Vázquez [5] suggested that effects of dual-task might be partially driven by intrusions from explicit memory and partially by intrusions from the secondary task information that disrupts the sequential stimulus organization (as in [7]). Across this extensive literature (21 published studies reviewed in [6]), the evidence points to a slowing in the learning rate under central executive constraints, but the effect size is difficult to disentangle from dual-task response timing effects.

Another persistent challenge of the dual-task paradigm is the possibility that the secondary task diverts perceptual attention away from the stimuli to be sequenced. Instructing participants to attend to the relevant stimulus dimensions affects whether sequence learning occurs [10] and can even protect participants from the disruption of a secondary task [11]. Thus, when using a dual-task paradigm, it is difficult to disentangle whether the central resource being constrained is the ability to attend to perceptual stimuli or the actual association rate of sequential elements during practice. To distinguish these possibilities, it will be necessary to use a resource constraint approach that does not require imposing dual-task conditions during learning.

A significant type of resource constraint for implicit learning may arise following the phenomenon of *ego depletion*. Muraven, Tice, and Baumeister [12] and Baumeister, Vohs, and Tice [13] proposed a strength model of cognitive control in which manipulations designed to deplete participants' reserve of cognitive control subsequently weakened central executive functioning in a range of contexts. Because cognitive control has been associated with variations in dopaminergic function [14], we hypothesized that *ego depletion* might reflect a transitory effect similar to the impairments in sequence learning observed in patients with Parkinson's Disease [15], [16]. A major advantage of using an ego depleting task to examine the effects of resource constraints on implicit sequence learning and performance is that the experimental manipulation can precede the learning or knowledge testing session and does not require imposition of a dual-task procedure.

To examine the effects of ego depletion on implicit learning, we used the Serial Interception Sequence Learning (SISL) task which produces robust implicit learning similar to the SRT task but with learning effects often detectable in individual participants [17]. While there is occasionally some concomitant explicit knowledge obtained by participants during practice, it has little or no effect on basic task performance [18] making the task effective for selectively examining implicit learning. Using a standard ego depletion manipulation [19], Experiment 1 revealed that ego depletion prior to skill learning led to poorer performance at test. In Experiment 2, ego depletion was examined pre-training and pre-test to attempt to disambiguate between learning and performance effects. A general reduction in skilled performance was again observed, although individual differences in effectiveness of the depletion manipulation precluded identifying differences based on administration timing. From the data obtained across both experiments, it appears that implicit skill learning, expression of acquired sequence knowledge, or perhaps both processes may depend on limited resources previously associated with cognitive control.

## Experiment 1 Method

### Participants

Thirty undergraduates (17 female, *M*_age_ = 20.1 years) at Northwestern University were compensated $10/hour for participation. This study was approved by Northwestern University's Institutional Review Board and participants provided written consent in accordance with IRB policy.

### Materials

#### Depletion task

All participants initially completed a 5 m, letter-crossing task in which a page of text was provided and they were instructed to cross out every letter “e”. For the next 5 m, Depletion condition participants completed a more complex, regulatory-control fatiguing task of crossing off every letter “e” unless it was next to or one letter removed from a vowel while Non-Depletion condition participants continued crossing out every letter “e” on the second page.

#### The Serial Interception Sequence Learning (SISL) task

**Figure 1:**
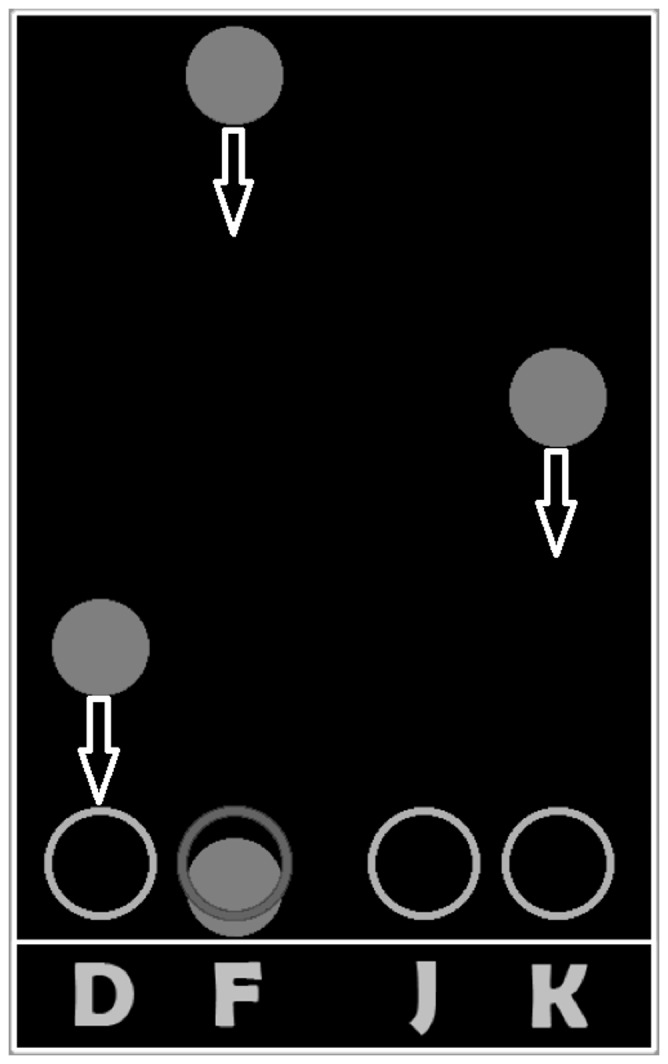
The Serial Interception Sequence Learning (SISL) task. Circular cues scroll down the computer screen (as indicated by the directional arrows) toward the target rings at the bottom. Participants respond when the circle is within the target ring by pressing the keyboard button indicated by the letter beneath the target ring.

Participants attempted to intercept cues scrolling down a monitor by pressing a corresponding key (D, F, J, K) when the cues overlapped target rings (Fig. 1). Responses were scored as correct if the corresponding key was pressed when the cue overlapped the target ring within one cue length (one half a cue length on either side of the optimal target response). The wrong key response, incorrect response timing, and multiple keypresses within one response window were all considered incorrect responses.

Cues initially scrolled down the screen at a velocity of 12.6 degrees/second, reaching the target zone .85 s after appearing on the screen. Speed was adapted based on performance, with cue velocity increasing when performance rose above 65% and dropping when performance fell below 25%. Participants were not told that cues followed a 12-item repeating sequence for 80% of the training trials; the other 20% of the trials were novel, non-repeating segments (more detail on the SISL task in [18]). All sequences were second-order conditional in structure [20].

### Procedure

Participants were randomly assigned to a Depletion (*n* = 15) or Non-Depletion (*n* = 15) condition. Three participants were excluded for low SISL task compliance (missed more than 30 trials within any single 60-trial block), resulting in 12 Depletion participants and 15 Non-Depletion participants for analysis. Participants first completed the Depletion tasks as described above, followed by a 2880-trail SISL task training session organized in six 480-trial blocks. Each training block contained 32 repetitions of the 12-item sequence and eight 12-item non-repeating sequences in a pseudo-random order. Following training, participants completed a 540-trial SISL test block to assess sequence-specific learning. The test block consisted of 15 repetitions of the trained sequence along with 15 repetitions of two novel SOC sequences in a pseudo-random order. Participants were then given a Recognition task, where they rated the trained sequence and four foils from -10 (definitely not seen) to +10 (definitely seen), with higher ratings for the trained sequence indicating some explicit knowledge.

## Experiment 1 Results

### SISL Task

#### SISL Training

A 2×6 mixed ANOVA of depletion condition (Depletion, Non-Depletion) and learning block (1–6) was used to examine learning of the trained sequence during training. Learning increased in a linear trend throughout training, *F*(1,25) = 28.87, *p*<.001, with the performance advantage for the repeating sequence compared to the foil sequences increasing from the first to last training block (Depletion, *M_block1_* = 0.74%, *SE_block1_* = 1.74%, *M_block6_* = 12.50%, *SE_block6_* = 2.88%; Non-Depletion, *M_block1_* = 0.16%, *SE_block1_* = 1.73%, *M_block6_* = 14.50%, *SE_block6_* = 2.69%). The interaction between depletion condition and learning block was not significant, *F*(5,125) = 0.79, *p* = .561, indicating that both conditions displayed reliable learning of the repeated sequence during the adaptive speed training.

#### SISL Test

**Figure 2:**
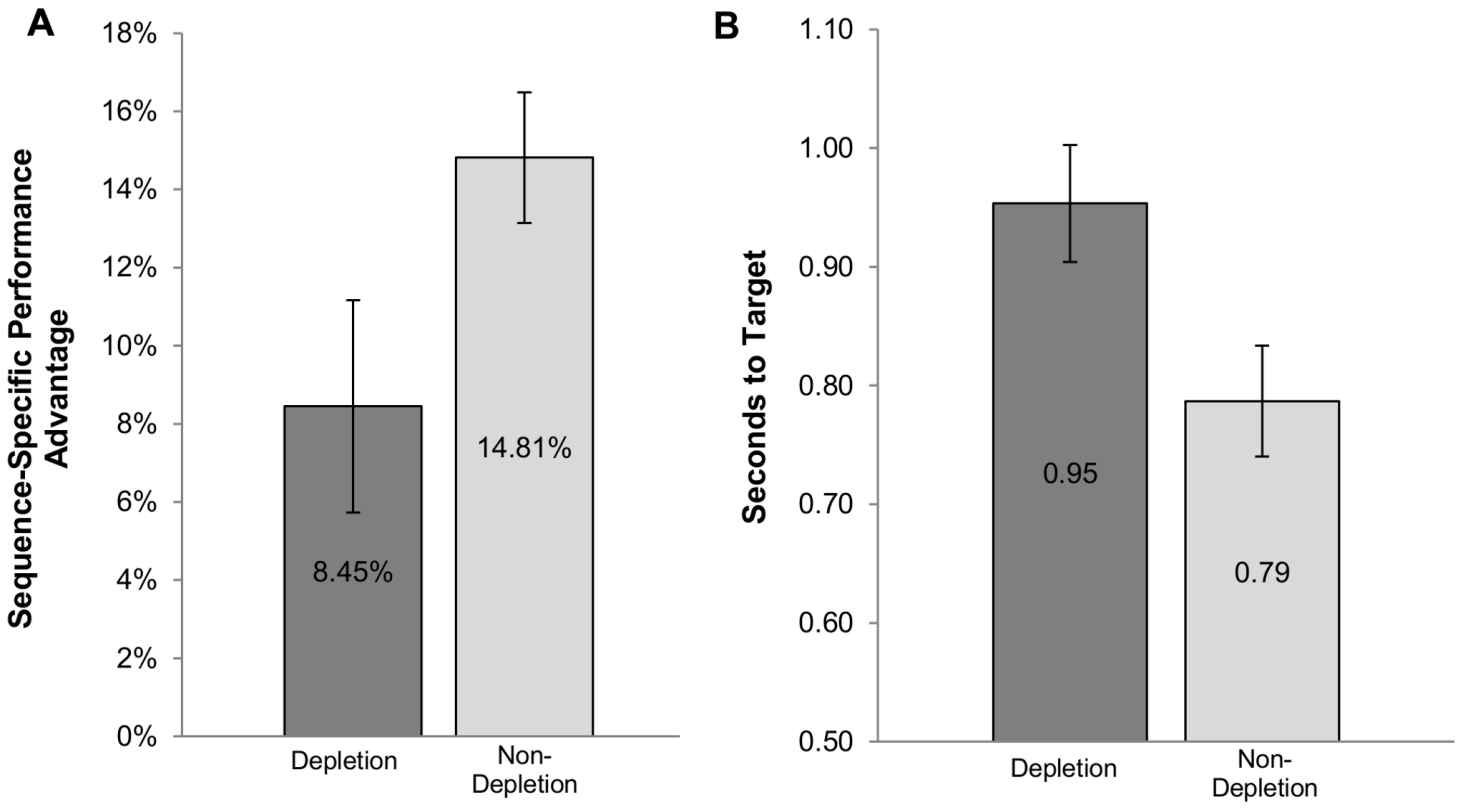
SISL test performance and speed for Experiment 1. The sequence specific performance advantage measures the improvement in SISL task execution when the cues are following the trained repeating sequence (calculated as the difference in percent correct for the trained sequence minus percent correct during untrained sequences of cues). (A) The Depletion group in Experiment 1 completed an ego-depleting task prior to training on the sequence. Less sequence-specific knowledge was expressed by the Depletion group at test. (B) Participants in the Depletion condition were also performing the task at test at a significantly slower rate at test than participants who had not been depleted (a higher number indicates the cues were taking longer to reach the target). The error bars reflect the standard error of the mean (SEM).

A 2×2 ANOVA of depletion condition (Depletion/Non-Depletion) and sequence type (trained/foils) assessed sequence-specific learning during test across groups. A main effect of sequence type reflected reliable sequence-specific knowledge in both groups, *F*(1,25) = 57.79, *p*<.001, with sequence blocks performed at higher accuracy than foil blocks (Figure 2). As expected, there was no main effect of depletion condition *F*(1,25) = 1.88, *p* = .183, due to the adaptive velocity that kept both groups performing at roughly 65% correct overall. However, a reliable interaction reflected a lower sequence-specific performance advantage in the Depletion group (*M* = 8.45%, *SE* = 2.72%) compared to the Non-Depletion group (*M* = 14.82%, *SE* = 1.67%), *F*(1,25) = 4.33, *p*<.05 (Fig. 2A). Participants in the Non-Depletion condition were also performing the SISL task at a much faster overall velocity at test (*M* = 0.79 seconds to target, *SE* = 0.05 s) than participants in the Depletion condition (*M* = 0.95 s, *SE* = 0.05 s), *t*(25) = 2.44, *p*<.05 (Figure 2B), suggesting an impairment in non-specific task elements for the Depletion group.

### Recognition Task

A 2×2 ANOVA of sequence type (trained/foils) and condition (Depletion/Non-depletion) for the recognition task revealed a main effect of sequence type, *F*(1,25) = 6.31, *p*<.05, indicating that participants gave higher recognition ratings to the trained sequence (*M* = 3.89, *SE* = .98) as compared to the foil sequences (*M* = .52, *SE* = .65). There was no main effect of depletion condition or any interaction (*Fs*<1).

### Discussion

Participants randomly assigned to the resource depleting condition prior to training exhibited a lower sequence-specific performance advantage and worse overall performance (i.e., slower speed) than those in the less depleting condition. The smaller sequence-specific benefit at test suggests that the implicit sequence learning rate may have been slowed during training, but it is also possible that the depletion effect persisted to and affected test performance. In Experiment 2, depletion was separately administered prior to training or prior to test and a subjective assessment of the depletion manipulation effect was used to measure how consistently participants were affected by the manipulation.

## Experiment 2 Method

### Participants

One hundred twenty-four participants (69 female, *M*_age_ = 19.2 years) at Northwestern University received course credit for participation. This study was approved by Northwestern University's Institutional Review Board and participants provided written consent in accordance with IRB policy.

### Materials

The SISL and Recognition tasks were identical to Experiment 1.

#### Depletion task

The Depletion manipulation was altered slightly for Experiment 2 to allow for the two time points (pre-training and pre-test) at which depletion could be administered. In the Non-Depletion task, participants were instructed to cross out every letter “e” on a page of text for 5 m and to write the sum of all e's they crossed off at the end of each line.

The Depletion manipulation involved crossing out “e's” on one page of text for 5 m, following the same, more complex, regulatory-control fatiguing rules as in Experiment 1. At the end of each line, participants tallied all the silent “e's” they had not crossed off. The text was printed several shades lighter than the Non-Depletion task, making it more difficult to read.

#### Depletion questionnaire

Participants provided self-reports of their own depletion levels after each encounter with the Depletion/Non-Depletion tasks. These ratings were made through a questionnaire with three 7-point scale ratings of task effort (“How much concentration did this task take?; “How effortful was it to cross off all the correct e's and provide a tally for each line?”) and mental state (“How tired are you now, compared to when you started this task?”). The sum of the three ratings was used as a self-report of general depletion level at each time point, with higher scores indicating that participants felt the task was more depleting.

### Procedure

Participants were randomly assigned to one of three conditions: Pre-training Depletion (*n* = 42), Pre-test Depletion (*n* = 41), or Non-Depletion (*n* = 41). Participants with low task compliance were excluded (6 Pre-training Depletion participants, 3 Pre-test Depletion participants, 7 Non-Depletion participants). There were two time points at which the depletion tasks could be administered: one before training on the SISL task, and one just before the SISL test block. The Pre-training Depletion group received the depleting form of the task prior to training and the non-depleting form of the task prior to test. The Pre-test Depletion group received the Non-Depletion task pre-training but completed the Depletion task prior to test. The Non-Depletion group completed the Non-Depletion form of the task at both time points.

## Experiment 2 Results

### SISL Task

#### SISL Training

A 3×6 mixed ANOVA of depletion condition (Non-Depletion, Pre-training Depletion, Pre-test Depletion) and learning block (1–6) was used to examine learning of the trained sequence during training. Learning increased in a linear trend throughout training (*F*(1,105) = .16, *p*<.001) with the performance advantage for the repeating sequence compared to the foil sequences increasing from the first to last training block (Non-Depletion, *M_block1_* = 5.73%, *SE_block1_* = 1.16%, *M_block6_* = 11.10%, *SE_block6_* = 1.70%; Pre-training Depletion, *M_block1_* = 4.78%, *SE_block1_* = 1.33%, *M_block6_* = 12.96%, *SE_block6_* = 1.47%; Pre-test Depletion, *M_block1_* = 3.54%, *SE_block1_* = 1.28%, *M_block6_* = 11.36%, *SE_block6_* = 1.67%). As in Experiment 1, the interaction between depletion condition and learning block was not significant (*F*(10,525) = 0.66, *p* = .767), suggesting that depletion condition did not affect measures of performance during the learning phase directly.

#### SISL Test

**Figure 3:**
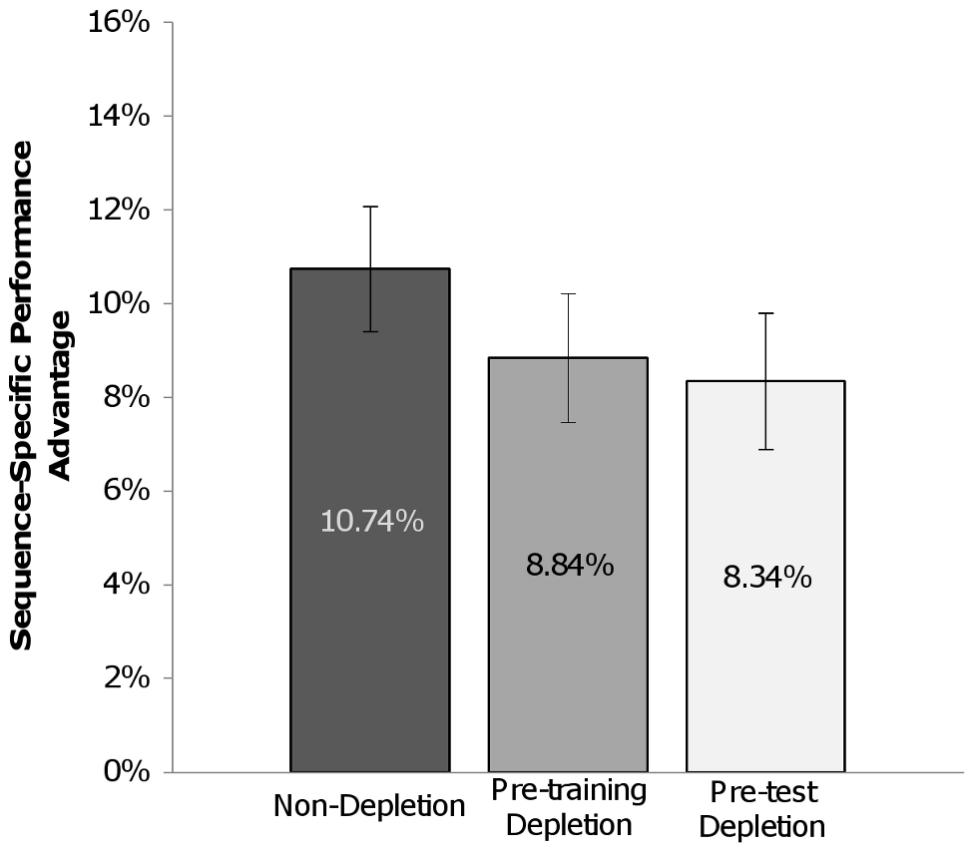
SISL test performance for Experiment 2. Participants who completed the challenging depletion task either prior to training (Pre-training Depletion) or prior to test (Pre-training Depletion) exhibited a smaller sequence-specific performance advantage at test compared to participants who had received the easy task (Non-Depletion). Error bars reflect SEM.

All three conditions exhibited better performance at test for the trained sequence than the unpracticed foil sequences, *F*(1,105) = 133.94, *p*<.001. There was no main effect of condition (Non-Depletion, Pre-training Depletion, Pre-test Depletion), *F*(2,105) = 1.19, *p* = .308, nor any interaction between condition and sequence type, *F*(2,105) = 0.81, *p* = .448. However, the performance benefit for the trained sequence compared to foil sequences was highest in the Non-Depletion group (*M* = 10.74%, *SE* = 1.33%), compared to both the Pre-training Depletion (*M* = 8.84%, *SE* = 1.38%) and Pre-test Depletion (*M* = 8.34%, *SE* = 1.45%) conditions (Fig. 3), suggesting that both groups who completed the Depletion task at some point expressed somewhat less sequence-specific knowledge.

Though the general pattern of results matched that of Experiment 1, the lack of a significant interaction between sequence type and condition suggested that the depletion manipulation may not have been as effective after it was altered to fit the two depletion time points. While participants in the Non-Depletion group rated the Non-Depletion task similarly at each time point (Pre-training, *M* = 9.44, *SE* = 0.44; Pre-test, *M* = 9.85, *SE* = 0.60), *t*(33) = .419, and lower than scores obtained following the Depletion task (*M* = 13.50, *SE* = 0.40 for the Pre-training Depletion group; *M* = 15.34, *SE* = 0.51 for the Pre-test Depletion group), *t*s>6.82, *p*s<.001, it was observed that quite a few individual participants reported depletion levels inconsistent with the experimental manipulation (e.g., reporting depletion after the non-depleting task or vice versa). Participants were therefore sorted post-hoc into depletion groups based on median split of self-reported depletion levels (D for higher scoring; ND for lower scoring) at the two manipulation time points. This yielded four different overall groups (ND-ND, *n* = 28; ND-D, *n* = 23; D-ND, *n* = 28; D-D, *n* = 29; Table 1). Analyses with these post-hoc groups are reported below.

**Table 1:** Post-hoc assignment of participants based on Depletion task self-rating and initial experimental condition.

	ND-ND	ND-D	D-ND	D-D
**Non-Depletion (** ***n*** ** = 36)**	20 (59%)	3 (9%)	4 (12%)	7 (20%)
**Pre-training Depletion (** ***n*** ** = 38)**	4 (11%)	1 (3%)	24 (67%)	7 (19%)
**Pre-test Depletion (** ***n*** ** = 34)**	4 (11%)	19 (50%)	0 (0%)	15 (39%)

Row labels refer to experimentally assigned conditions, while column labels denote groupings based on self-reported depletion task ratings. Across columns, D/ND refers to self-reported depletion task rating (above/below median score) just prior to training and just prior to test. The four possible conditions are: ND-ND, not reporting as depleted by the letter-crossing task at either time point; ND-D, reporting as depleted by the task prior to test but not training; D-ND, reporting as depleted by the task prior to training but not test; and D-D, reporting as depleted by the task at both time points. Across rows are the experimentally assigned conditions: Non-Depletion participants performed the easier, non-depleting task prior to both training and test; Pre-training Depletion participants performed the depleting task prior to training and the easier task before test; Pre-test Depletion participants performed the depleting task before test. This post-hoc grouping identifies participants who appear to have recovered from depletion over the course of learning (e.g., the 24 Pre-training Depletion participants who fell into the D-ND post-hoc group) as well as participants who may have been depleted by the SISL task itself (e.g., the 10 participants in the Non-Depletion condition who reported depletion prior to test; ND-D or D-D) or come into the experiment with high levels of depletion (e.g., the 15 participants in the Pre-test Depletion condition who already self-reported feeling depleted prior to training).

### Self-Rated Depletion

**Figure 4:**
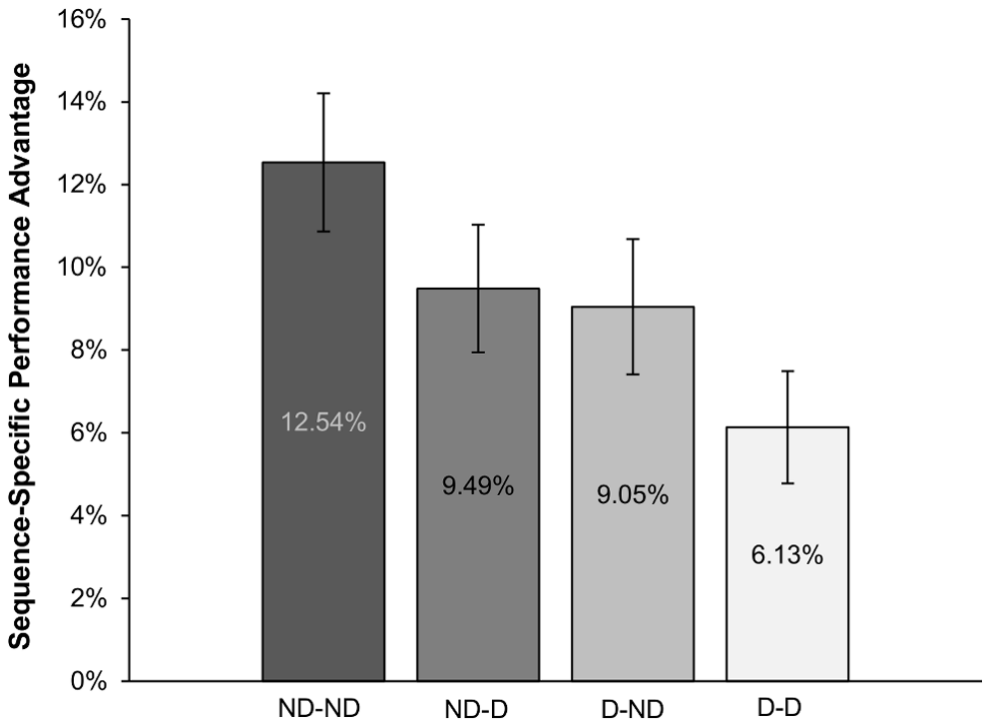
SISL test performance for post-hoc conditions in Experiment 2. Participants in a depleted state prior to training and test (D-D) exhibited a significantly smaller sequence-specific performance advantage at test compared to those who were not depleted at either time point (ND-ND). Those who self-reported depletion at either time point (ND-D and D-ND groups) also displayed reduced performance benefits compared to ND-ND participants. Error bars reflect SEM.

The post-hoc grouping allows for a 2×2 ANOVA of depletion and time point (pre-training/pre-test) that groups participants by their self-reported (actual) depletion level rather than the intended level (experimental assignment to groups). With the magnitude of the sequence-specific performance benefit as the measure of expression of sequence learning, this analysis revealed a main effect of depletion Pre-training, *F*(1,104) = 4.76, *p*<.05, a marginal effect of depletion Pre-test, *F*(1,104) = 3.62, *p* = .060 and no interaction between conditions, *F*(1,104) = 0.002, *p* = .966. Accordingly, participants who did not report feeling depleted at either time point (ND-ND) showed a typical performance advantage for the trained sequence (*M* = 12.54%, *SE* = 1.67%), similar to that of Non-Depletion participants in Experiment 1. Participants with no self-reported depletion prior to training who subsequently reported feeling depleted before test (ND-D) showed slightly impaired performance (*M* = 9.49%, *SE* = 1.54%), as did participants with the opposite depletion pattern (D-ND; *M* = 9.05%, *SE* = 1.63%). Finally, participants who reported feeling depleted at both time points (D-D) had the lowest overall sequence benefit (*M* = 6.13%, *SE* = 1.36%), with a significantly lower sequence-specific benefit than the ND-ND group, *p*<.05 (Fig. 4). There were no main effects of post-hoc depletion condition on SISL test performance speed, *F*s<1.39, *p*s>.24 or explicit sequence knowledge, *F*s<1.98, *p*s>.12.

### Recognition Task

Participants showed higher recognition for the trained sequence (trained sequence, *M* = 4.38, *SE* = .54; foil sequences, *M* = −.99, *SE* = .40), *F*(1,105) = 59.83, *p*<.001. There was no main effect of depletion condition on recognition knowledge or any interaction (*Fs*<1).

### Discussion

Participants who were experimentally assigned to receive the cognitive resource depletion manipulation at any point exhibited numerically less sequence-specific knowledge at test than those who were not depleted, following the basic pattern from Experiment 1. Though the effect was not as strong, this was likely due to shortening of the time participants were required to perform the regulatory fatiguing task before training, perhaps suggesting a dose-dependent effect whereby more depletion leads to greater impairment. When participants were grouped based on severity of self-rated depletion estimates, a clear effect was observed with depletion leading to a smaller sequence-specific performance advantage at test. Thus, across both experiments, ego depletion led to impaired sequence performance.

In addition, a goal of Experiment 2 was to attempt to separate the effects of depletion prior to training and prior to test in order to delineate effects due to sequence learning or expression, respectively. However, any evidence of depletion during the training phase of the protocol, as measured by higher self-rated depletion score either pre- or post-training, was associated with poorer sequence-specific performance at test. These effects were additive, with an even greater impairment observed in participants reporting depletion at both time points. While the effects of depletion were observed at test in both experiments, in Experiment 2, participants who only self-reported depletion prior to training (the D-ND group) also exhibited lower sequence-specific knowledge. Thus we can conclude that depletion affects either or possibly both of the processes of (1) acquiring implicit knowledge or (2) the stimulus-specific expression of that knowledge.

## General Discussion

Implicit learning has often been described as an automatic process that should be resistant to effects of reducing central processing resources. Previous studies using dual-task methodologies have suggested that resource constraints affect learning, but the logistics of a dual-task protocol left it unclear whether this was due to reductions in available executive processes, disruption in organizational processes, dispersal of attention, or measurement issues related to the timing of main task and secondary task responses [5], [6], [8], [9]. Here, executive resources were reduced prior to implicit learning using an ego depletion manipulation designed to fatigue a limited store of cognitive control resources. This type of manipulation has been shown to generate effects specifically related to weakened central executive functioning, which in turn impairs cognitive functions such as choice behavior and self-regulation [12], [13]. In the two experiments reported here, this ego depletion was found to lead to impaired expression of sequence-specific knowledge. Participants who were depleted prior to or during learning exhibited less implicit knowledge as measured by the magnitude of the sequence-specific performance advantage expressed on the post-training test.

Prior research on reduction of executive functions during implicit learning has suggested that constraining central resources affects learning rather than performance [6], [8], [22]. Prior reports have also suggested that ego depletion affects complex cognitive processes more than well-learned skills [21]. Our results could potentially reflect a novel effect of ego depletion specifically on sequence-specific performance at test. Participants who self-reported depletion only immediately before (D-ND) or after training (ND-D) both exhibited less sequence knowledge on the post-training SISL test. Thus, ego depletion may affect implicit learning, expression of implicitly acquired knowledge or even both processes.

The measure of knowledge in the SISL task is a comparison of performance for the trained and untrained sequences. As a result, the potential general effect of ego depletion on non-specific aspects of performance (e.g., general slowing due to fatigue) should not affect the measure of implicit knowledge. Rather, the reduction in resources accomplished via the depletion manipulation affected performance of the specific, trained sequence. It appears that the ego depletion manipulation affects the neurocognitive processes either associated with extracting the hidden repeating sequential structure, or applying this knowledge later when the sequence is re-encountered.

Explicit memory for the repeating sequence was not found to be affected by the depletion manipulation in either experiment arguing against the possibility raised by Jiménez & Vázquez [5] that resource constraints affect some implicit learning measures via effects on explicit knowledge that can contaminate some implicit tasks. Although participants exhibited some ability to recognize the trained sequence here, the SISL task is particularly resistant to explicit memory effects on implicit learning performance. Even full explicit sequence knowledge does not providing a measurable performance benefit [18]. While using the SISL task allowed for a selective assessment of the effect of resource constraints on implicit learning, the effect of resource depletion on more complex tasks in which implicit and explicit strategies are both available will need to be examined further. For example, Filoteo, Lauritzen, and Maddox [23] found that performance on an implicit category learning task actually improved under distraction conditions that encouraged reliance on the optimal task strategy. Based on the current results, we would suggest that distraction likely slowed both implicit and explicit learning but that the slowing of implicit category learning was a smaller cost than the benefit of discouraging reliance on the suboptimal explicit strategy.

Given that implicit learning occurs outside of awareness, the dependence on central executive resources seems somewhat counter-intuitive. A potential mechanism for this effect is through transient variation in dopaminergic functioning imposed by depletion. Disruptions in dopaminergic function have been associated with impaired sequence learning [15], [16], [24], [25]. These studies have typically examined learning in patients with Parkinson's disease (PD) and indicate the importance of dopamine-gated plasticity in perceptual-motor sequence learning. Milder manipulations in dopaminergic function have also been shown to affect cognitive processing [26] and these can be observed in individual differences as well [27]. The neuropharmacological basis of ego depletion has not been explored, but it is notable that manipulations that alleviate depletion effects (e.g. mood enhancement, surprise gifts; [28]) likely involve increased dopaminergic availability. If the ego depleting task transiently reduces dopaminergic levels in participants, the subsequent impairment of skilled performance might occur due to the same disruption in dopamine-gated plasticity that is observed in PD. By this mechanism, individual differences in dopaminergic function might lead to individual differences in learning rate like those reported in Kaufman, et al. [29] as well as the performance deficits we observed. In addition, these might interact with other contextual factors such as pre-existing life stressors experienced either before an experimental session or a non-laboratory skill learning experience.

The adverse effect of depletion on skilled performance suggests that it is important to maximize central processing resources to get the greatest benefit from practice during skill learning through repetitive practice and support later performance. While the SISL task primarily measures implicit perceptual-motor skill learning, common mechanisms are hypothesized to support skill learning across a broad range of domains, including cognitive skills [30]. Maximizing the gains from training with repeated practice in contexts from cognitive to physical skills will depend on understanding both the effects of changes in executive processing resources and the role of the brain's multiple memory systems in the task being learned.

## Supporting Information

Data S1 **Depletion data.**
(ZIP)
